# Activation of the Hippo Pathway by ATN Suppresses Cancer Stem-Like Properties and EGFR-Associated Signaling in Castration-Resistant Prostate Cancer Cells *in vitro*

**DOI:** 10.7150/ijms.129402

**Published:** 2026-05-01

**Authors:** Kuan-Jung Lin, Jui-Ming Liu, Hsiang-Wen Lin, Jing-Ru Weng, Eman M. E. Dokla, Meng-Tien Lu, Po-Chen Chu

**Affiliations:** 1 Division of Urology, Department of Surgery, Taoyuan General Hospital, Ministry of Health and Welfare, Taoyuan 33004, Taiwan.; 2 Department of Urology, College of Medicine and Shu-Tien Urological Research Center, National Yang Ming Chiao Tung University, Taipei 112304, Taiwan.; 3 Department of Obstetrics and Gynecology, Tri-Service General Hospital, National Defense Medical Center, Taipei 114202, Taiwan.; 4 School of Pharmacy, College of Pharmacy, China Medical University, Taichung 406040, Taiwan.; 5 Department of Pharmacy, China Medical University Hospital, Taichung 406040, Taiwan.; 6 Department of Marine Biotechnology and Resources, National Sun Yat-sen University, Kaohsiung 80424, Taiwan; 7 Graduate Institute of Natural Products, Kaohsiung Medical University, Kaohsiung 80708, Taiwan.; 8 Graduate Institute of Pharmacognosy, College of Pharmacy, Taipei Medical University, Taipei 11031, Taiwan.; 9 Pharmaceutical Chemistry Department, Faculty of Pharmacy, Ain Shams University, Abbassia, Cairo 11566, Egypt.; 10 Department of Cosmeceutics and Graduate Institute of Cosmeceutics, China Medical University, Taichung 406040, Taiwan.

**Keywords:** castration-resistant prostate cancer, YAP, epidermal growth factor receptor (EGFR), amphiregulin (AREG), cancer stem cells, Akt/mTOR

## Abstract

**Background:**

Castration-resistant prostate cancer (CRPC) represents a lethal stage of prostate cancer for which effective therapeutic options remain limited. Aberrant activation of the Hippo pathway effector Yes-associated protein (YAP) and its crosstalk with epidermal growth factor receptor (EGFR) signaling have been implicated in tumor progression, cancer stemness, and metastasis. This study aimed to investigate the therapeutic potential and underlying mechanisms of ATN, a 1,2,4-oxadiazole-based YAP inhibitor, in CRPC cells.

**Methods:**

The anti-cancer activity of ATN was evaluated in CRPC cell lines, PC3 and DU145, using cell viability, colony formation, apoptosis, tumorsphere, and transwell migration assays. Western blotting and immunofluorescence analyses were performed to assess Hippo-YAP and EGFR signaling pathways. Functional rescue experiments were conducted using YAP overexpression and constitutively active Akt (CA-Akt).

**Results:**

ATN significantly suppressed CRPC cell viability in a concentration-dependent manner, with IC₅₀ values of 1.13 μM for PC3 cells and 0.43 μM for DU145 cells, while exhibiting lower cytotoxicity toward non-tumorigenic fibroblasts (IC₅₀ = 1.72 μM). Mechanistically, ATN increased YAP phosphorylation at Ser397, promoted its degradation, and reduced nuclear YAP localization. This was accompanied by downregulation of YAP target proteins, including c-Myc and NFκB. Functionally, ATN significantly inhibited proliferation, induced apoptosis, and suppressed tumorsphere formation and CD44^+^/CD24^-^ cancer stem cell (CSC) population in CRPC cells. In addition, ATN inhibited cell migration and reduced EMT-related proteins, including N-cadherin, Twist, YB-1, and Snail. ATN also decreased amphiregulin (AREG) expression, leading to suppression of EGFR activation and downstream MEK/ERK and Akt/mTOR signaling pathways. Importantly, constitutive activation of Akt partially rescued ATN-induced cytotoxicity (~78% recovery), confirming the involvement of YAP-associated EGFR/Akt signaling.

**Conclusion:**

ATN exerts potent anti-tumor effects against CRPC cells by activating the Hippo pathway, promoting YAP degradation, and disrupting the YAP-associated EGFR signaling network. These findings highlight ATN as a promising therapeutic candidate for targeting YAP-driven oncogenic signaling in CRPC.

## Introduction

Prostate cancer is one of the most frequently diagnosed malignancies in men and remains a major contributor to cancer-related mortality worldwide. According to global cancer statistics, prostate cancer accounted for approximately 1.5 million new cases and nearly 397,000 deaths in 2022, ranking as the second most common cancer and the second leading cause of cancer death in men 1. Surgery and radiation therapy are the standard therapies for early-stage or localized prostate cancer, with a 5-year survival rate nearly 100%. In contrast, androgen deprivation therapy (ADT) represents the first-line treatment for locally advanced or metastatic patients 2. Although the majority of patients initially respond to ADT, resistance invariably develops within 1-3 years, resulting in the emergence of castration-resistant prostate cancer (CRPC), a stage associated with poor prognosis and markedly reduced overall survival 3. Metastatic CRPC remains incurable, underscoring an urgent need for the development of novel therapeutic strategies to improve clinical outcomes in this lethal disease.

The Hippo signaling pathway serves as an evolutionarily conserved regulator of organ size, tissue homeostasis, regeneration, cellular proliferation, survival, and differentiation 4. The Hippo pathway is mainly composed of mammalian Ste20-like kinases 1/2 (MST1/2), SAV1, MOB kinase activator 1A/B (MOB1A/B), large tumor suppressor kinase 1/2 (LATS1/2), Yes-associated protein (YAP) and its paralog, transcriptional coactivator with a PDZ-binding motif (TAZ) 5. YAP, one of the core effectors of the Hippo pathway, acts as a transcriptional co-activator involved in regulating embryonic development, organ growth, tissue development, homeostasis, and regeneration 6. The activity of YAP protein is negatively regulated by the phosphorylation cascades in Hippo pathway. In mammalian cells, when the Hippo pathway is active, the kinase cascade involving MST1/2, SAV1, MOB1A/B, and LATS1/2 phosphorylates YAP, leading to its cytoplasmic retention and degradation by the ubiquitin proteasome pathway. In contrast, when the Hippo pathway is inactive, unphosphorylated YAP translocates into the nucleus to promote transcription of genes associated with cell growth, survival, and proliferation via interactions with TEAD1-TEAD4 transcription factors 7.

Dysregulation of the Hippo pathway has been shown to be involved in tumorigenesis and metastasis in several cancers, including lung, colorectal, breast, ovarian, liver, and prostate cancer 8, 9. Elevated expression and nuclear accumulation of YAP have been detected in multiple malignancies, including brain, breast, pancreatic, ovarian, lung, liver, gastric, and prostate cancers, correlating with aggressive disease behavior and poor survival 10, 11. Constitutive activation of YAP has also been reported to promote tumor initiation, progression, metastasis, maintenance of cancer stem cells, and resistance to therapies 6. Within prostate cancer, mounting evidence supports a pivotal oncogenic role for YAP. Increased YAP expression associates with extraprostatic extension of prostate cancer and may serve as a prognostic indicator of disease progression 12-14. Ectopic expression of YAP promoted prostate cancer cell motility, invasion, and tumor growth of CRPC 12. Targeting YAP via pharmacologic inhibitor, verteporfin, or genetic knockdown was able to inhibit the castration-resistant human xenograft prostate tumor growth and induce apoptosis in prostate cancer cells, respectively 15. Collectively, these findings provide a compelling rationale for targeting YAP as a therapeutic strategy in prostate cancer, particularly for addressing the unmet clinical need for CRPC.

The EGFR signaling axis plays a central role in regulating cell proliferation, survival, differentiation, and migration, and its dysregulation has been extensively implicated in the initiation and progression of prostate cancer 16-18. Aberrant activation of EGFR and its downstream pathways, including the PI3K/Akt/mTOR and RAS/MEK/ERK cascades, contributes to uncontrolled growth, resistance to apoptosis, and therapeutic resistance in advanced prostate cancer 19, 20. Elevated EGFR expression and ligand availability have been correlated with disease progression, epithelial-mesenchymal transition (EMT), metastasis, cancer stemness traits, and resistance to androgen-deprivation therapy, thereby contributing to the lethal phenotype of CRPC 21, 22. Emerging evidence has indicated that YAP can regulate the EGFR pathway, thereby reinforcing oncogenic signaling and contributing to aggressive phenotypes 23, 24. These findings underscore the critical role of the EGFR axis in prostate cancer biology and support the rationale for therapeutic strategies targeting YAP-EGFR cross-talk in prostate cancer. Recent evidence further supports an important role for YAP-receptor tyrosine kinase (RTK) crosstalk in advanced prostate cancer. In CRPC, Traf2- and Nck-interacting kinase (TNIK) has been shown to activate EGFR under androgen-deprived conditions, highlighting how alternative kinase inputs can sustain RTK signaling during therapy resistance 21. Collectively, these findings support the view that YAP-RTK cooperation is an important feature of CRPC progression and further justify therapeutic approaches aimed at disrupting this network 25, 26.

Pursuant to our previous findings, ATN (Figure 2A), a novel 1,2,4-oxadiazole derivative, has emerged as a promising modulator of Hippo-YAP signaling. Previous studies demonstrated that ATN promotes YAP degradation through activation of upstream Hippo kinases, particularly LATS1/2, thereby restoring Hippo pathway activity. Functionally, ATN has been shown to exert potent anti-tumor effects in breast cancer by suppressing tumor growth and to induce apoptosis in oral squamous cell carcinoma cells through YAP destabilization 27, 28. Although several YAP-targeting strategies have been proposed, their clinical translation remains limited. The best-characterized YAP inhibitor, verteporfin, suppresses YAP-TEAD transcriptional activity and has shown anti-tumor effects in preclinical models; however, its broader application in cancer therapy is constrained by phototoxicity, poor bioavailability, and off-target effects. In addition, many other YAP-targeting approaches under preclinical development act indirectly on upstream regulators or disrupt YAP-TEAD interaction, but often suffer from limited selectivity, insufficient potency, or suboptimal pharmacokinetic properties. These limitations highlight the need to identify alternative YAP-targeting agents with distinct mechanisms of action and improved therapeutic potential. Notably, the mechanism of ATN differs from that of the well-characterized YAP inhibitor verteporfin, which primarily disrupts YAP-TEAD transcriptional activity without directly inducing YAP degradation. In contrast, ATN functions as a Hippo pathway activator that enhances YAP phosphorylation and promotes its proteasomal degradation, thereby leading to sustained suppression of YAP signaling. This distinct pharmacological profile suggests that ATN may provide advantages over existing YAP-targeting strategies by targeting upstream regulatory mechanisms rather than solely interfering with transcriptional complex formation. Despite these promising findings, the therapeutic potential and mechanistic role of ATN in prostate cancer, particularly in CRPC, remain unexplored. Given the critical role of YAP in CRPC progression and its functional interaction with EGFR signaling, we hypothesized that ATN may exert anti-cancer effects in CRPC by destabilizing YAP and disrupting YAP-associated EGFR oncogenic signaling. In the present study, we aimed to investigate the therapeutic potential and underlying molecular mechanisms of ATN in CRPC cellular models. Specifically, we examined whether ATN-mediated Hippo pathway activation leads to YAP degradation and subsequent suppression of EGFR downstream pathways, thereby inhibiting proliferation, cancer stem-like properties, migration, and survival of CRPC cells. By addressing this previously unexplored context, this study seeks to establish ATN as a novel YAP-targeting agent and to provide mechanistic insights into the YAP-EGFR signaling axis in CRPC.

## Materials and Methods

### Cell lines, cell culture, and antibodies

Human prostate cancer cell lines, PC3 (Cat#60122), DU145 (Cat#60348), LNCaP (Cat#60088), and 22Rv1 (Cat#60545), were obtained from the Bioresource Collection and Research Center (BCRC, Hsinchu, Taiwan). The human non-tumorigenic skin fibroblast CCD966SK cell line (BCRC, Hsinchu, Taiwan, Cat#60153) was a kind gift from Prof. Louis Kuoping Chao (China Medical University, Taichung, Taiwan). All cells were cultured in RPMI 1640 medium (Thermo Fisher Scientific, Carlsbad, CA, USA, Cat#23400021) with 10% fetal bovine serum (FBS) (Thermo Fisher Scientific, Cat#A5670701) and 1% Penicillin/Streptomycin (Thermo Fisher Scientific, Cat#15140122) at 37 °C in a humidified incubator containing 5% CO2. Recombinant human EGF was obtained from Sino Biological (Beijing, China, Cat#10605-HNAE). The YAP (1:1000, Cat#14074), c-Myc (1:1000, Cat#5605), NFκB (1:1000, Cat#8242), p-S397-YAP (1:1000, Cat#13619), p-T180/T183-MST1/2 (1:1000, Cat#49332), MST1 (1:1000, Cat#3682), p-T1079-LATS1 (1:1000, Cat#8654), LATS1 (1:1000, Cat#3477), PARP-1 (1:1000, Cat#9542), Caspase 9 (1:1000, Cat#9502), Caspase 3 (1:1000, Cat#9662), Oct4 (1:1000, Cat#2750), SOX2 (1:500, Cat#3579), Nanog (1:500, Cat#4903), YB-1 (1:2000, Cat#4202), Snail (1:1000, Cat#3879), p-EGFR (1:1000, Cat#3777), p-MEK (1:2000, Cat#9154), MEK (1:1000, Cat#8727), p-ERK (1:2000, Cat#4370), ERK (1:1000, Cat#4695), p-S473-Akt (1:1000, Cat#4060), p-T308-Akt (1:1000, Cat#13038), Akt (1:1000, Cat#9272), p-mTOR (1:1000, Cat#5536), mTOR (1:1000, Cat#2983), and p-p70S6K (1:1000, Cat#9205) antibodies were purchased from Cell Signaling Technology (Beverly, CA, USA). The Flag (1:5000, Cat#F1804) antibody was purchased from Sigma-Aldrich (St. Louis, Mo, USA). The β-Actin (1:5000, Cat#sc-47778), N-cadherin (1:1000, Cat#sc-59987), Twist (1:1000, Cat#sc-81417), EGFR (1:1000, Cat#sc-373746), Amphiregulin (1:500, Cat#sc-74501), and p70S6K (1:1000, Cat#sc-8418) antibodies were purchased from Santa Cruz Biotechnology (Santa Cruz, CA, USA). The CD44-PE (1:50, Cat#12-0441-82) and CD24-APC (1:50, Cat#17-0242-82) antibodies were purchased from Thermo Fisher Scientific (Carlsbad, CA, USA). The CD133 (1:1000, Cat#GTX32496) and CD44 (1:1000, Cat#GTX637231) antibodies were purchased from GeneTex (Irvine, CA, USA). The GAPDH (1:5000, Cat#60004-1-Ig) antibody was purchased from ProteinTech (Chicago, IL, USA). The HRP-Goat Anti-mouse IgG antibody (1:2000, Cat#115-035-146) and HRP-Goat Anti-rabbit IgG antibody (1:2000, Cat#111-035-144) were purchased from Jackson ImmunoResearch Laboratories (West Grove, PA, USA).

### Reagents

ATN, {[N-(4-(5-(3-(3-(4-acetamido-3-[trifluoromethyl]phenyl)ureido)phenyl)-1,2,4-oxadiazol-3-yl)-3-chlorophenyl)-nicotinamide]}, was synthesized and characterized as previously reported 27, following established procedures for 1,2,4-oxadiazole derivatives. The purity (98.37%) of ATN was determined by HPLC, and its chemical identity was confirmed by ¹H NMR, ¹³C NMR, and high-resolution electrospray ionization mass spectrometry (ESI-HRMS) analyses prior to biological evaluation 27.

### Cell viability assay

The ATN stock solution (5 mM) was freshly prepared in DMSO (Sigma-Aldrich, Cat#D8418) and diluted with culture medium to the indicated concentrations with a final DMSO concentration of 0.1%. Prostate cancer cells were plated into 96-well culture plates at a density of 5×10^3^ cells per well using growth medium supplemented with 10% FBS. Following an overnight attachment period, the medium was replaced with 5% FBS medium containing indicated concentrations of ATN or DMSO control, and cultures were maintained for 72 hours. To assess cell viability, 3-(4,5-dimethylthiazol-2-yl)-2,5-diphenyltetrazolium bromide (MTT) solution (Biomatik, Wilmington, DE, USA; Cat#A3338) was added and incubated for 1 h. After removing the supernatant, the formazan crystals formed were dissolved in DMSO, and absorbance was determined at 570 nm using a microplate reader. Cell viability was calculated as the percentage of absorbance relative to the DMSO-treated control wells.

### Transient transfection

The wild-type YAP-Flag expression plasmid (plasmid #66853) and constitutively active Akt plasmid (CA-Akt) (plasmid #10841) were obtained from Addgene (Watertown, MA, USA). The scrambled siRNA was purchased from Dharmacon (Horizon Discovery, Cat#D-001206-13-05) and YAP siRNA was purchased from Santa Cruz Biotechnology (Santa Cruz, Cat#sc-38637). The plasmid or siRNA was introduced into cells using Lipofectamine 2000 (Thermo Fisher Scientific, Cat#11668019) following the manufacturer's instructions. Twenty-four hours post-transfection, cells were reseeded at a density of 5,000 cells per well in 96-well plates, and cell viability was subsequently determined using the MTT assay.

### Western blot analysis

Following ATN treatment, cells were collected, rinsed with ice-cold PBS, and lysed in SDS buffer supplemented with protease and phosphatase inhibitors (Thermo Fisher Scientific, Cat#78440). Total protein content was quantified using the BCA assay (Thermo Fisher Scientific, Cat#23227). Equal amounts of protein were resolved on 8-12% SDS-polyacrylamide gels and subsequently transferred onto nitrocellulose membranes (Sigma-Aldrich, Cat#GE10600002). Membranes were blocked for 1 h in TBST (TBS with 0.1% Tween-20) containing 5% nonfat milk (Sigma-Aldrich, Cat#M7409), then incubated overnight at 4°C with the indicated primary antibodies. After washing with TBST, membranes were probed with appropriate HRP-conjugated secondary antibodies for 1 h at room temperature. Immunoreactive proteins were visualized using Chemiluminescence Reagent Plus (PerkinElmer, Waltham, MA, USA, Cat#PK-NEL105). The values indicated beneath the immunoblots represent fold changes calculated by normalizing band intensities of treated samples to the internal reference protein GAPDH or β-Actin and expressing them relative to the corresponding vehicle-treated controls. Densitometric analysis of protein expression was performed using ImageJ software (version 1.52a).

### Colony formation assay

Cells were plated into six-well culture dishes at a density of 1 × 10⁴ cells per well and allowed to adhere overnight. The following day, cells were exposed to ATN at the indicated concentrations and maintained for 7-14 days until macroscopically visible colonies developed. Colonies were then fixed in 4% formaldehyde (Sigma-Aldrich, Cat#252549) and stained with crystal violet solution (Sigma-Aldrich, Cat#C6158). Only colonies containing > 50 cells were included in the analysis. Colony numbers were quantified and expressed as a percentage relative to vehicle-treated controls.

### Apoptosis assays by flow cytometry

Apoptotic populations were assessed using the Annexin V-FITC Apoptosis Detection Kit (BD Pharmingen, San Diego, CA, USA; Cat#556547). Human CRPC cells were exposed to ATN at the indicated concentrations or to vehicle control (DMSO) for 72 hours. After treatment, cells were collected, washed, and incubated in binding buffer containing Annexin V-FITC and propidium iodide (PI) for 20 min at room temperature in the dark. Samples were subsequently analyzed on a BD FACSCanto™ flow cytometer (BD Biosciences, Franklin Lakes, NJ, USA), and the proportions of apoptotic cells were quantified from the acquired data. The apoptotic cells were defined as the sum of Annexin V-FITC positive/PI negative (early apoptosis) and Annexin V-FITC positive/PI positive (late apoptosis) populations.

### Immunofluorescence staining

Cells were seeded onto coverslips overnight in 6-well culture plates. After treatment with ATN, cells were fixed with 4% paraformaldehyde for 20 minutes, and then permeabilized with 0.2% Triton X-100 for 10 minutes. These cells were blocked with 1% BSA in PBS for 30 minutes, and then incubated with primary antibody (YAP, 1:100, Cell Signaling Technology, Cat#14074) overnight at 4 °C. After washing with PBS, cells were incubated with secondary antibody conjugated with Alexa Fluor 488 (1:100, Thermo Fisher Scientific, Cat#A-11008) for 1 hour at 37 °C. The cover glasses were stained with 4',6-diamidino-2-phenylindole (DAPI, Sigma-Aldrich, Cat#D9542) for nuclear staining and then mounted with Fluoromount Mounting Medium (Sigma-Aldrich, Cat#F4680). The images were captured under a fluorescence microscope (Nikon ECLIPSE 80i, Nikon, Japan) equipped with a DS-Qi1MC CCD camera (Nikon).

### Cancer stem cells tumorsphere formation assay

To evaluate the influence of ATN on CRPC cell stemness, cells were seeded at a density of 5000 cells per well into ultra-low attachment 24-well plates (Corning Inc., Union City, CA, USA; Cat#3473) and maintained in MammoCult™ Human Medium (STEMCELL Technologies, Vancouver, BC, Canada; Cat#05620) with the indicated concentrations of ATN or vehicle control (DMSO). Cultures were maintained for 8-12 days, during which tumorsphere growth was monitored. At the endpoint, spheres were visualized and imaged using a light microscope at 100× magnification. The number of tumorspheres was quantified using trypan blue exclusion.

### Transwell cell migration assay

The migratory capacity of CRPC cells in response to ATN was examined using transwell chambers fitted with 8-µm pore membranes (Millipore, Bedford, MA, USA; Cat#SCWP09025). Cells were resuspended in 300 μL of serum-free medium containing the indicated concentrations of ATN and seeded into the upper compartment of each chamber. The lower wells were filled with 800 μL of medium supplemented with 10% FBS (Thermo Fisher Scientific, Cat#A5670701) to serve as a chemoattractant. After incubation at 37 °C for 16-24 hours, cells remaining on the upper surface of the membrane were gently removed with a cotton swab. The membranes were then fixed with methanol, stained using Giemsa solution (Sigma-Aldrich, Cat#32884), and visualized under a light microscope at 100× magnification. Migrated cells on the underside of the membrane were counted in randomly chosen fields, and results were expressed as percentages relative to the DMSO-treated control group.

### Statistical analysis

All quantitative results are expressed as mean ± standard deviation (S.D.). Differences between two experimental groups were evaluated using Student's t-test. For comparisons involving multiple groups, two-way ANOVA followed by Sidak's post hoc test was applied. P-value < 0.05 was considered statistically significant.

## Results

### YAP is highly expressed in prostate cancer cells and contributes to cell proliferation

**Figure 1:**
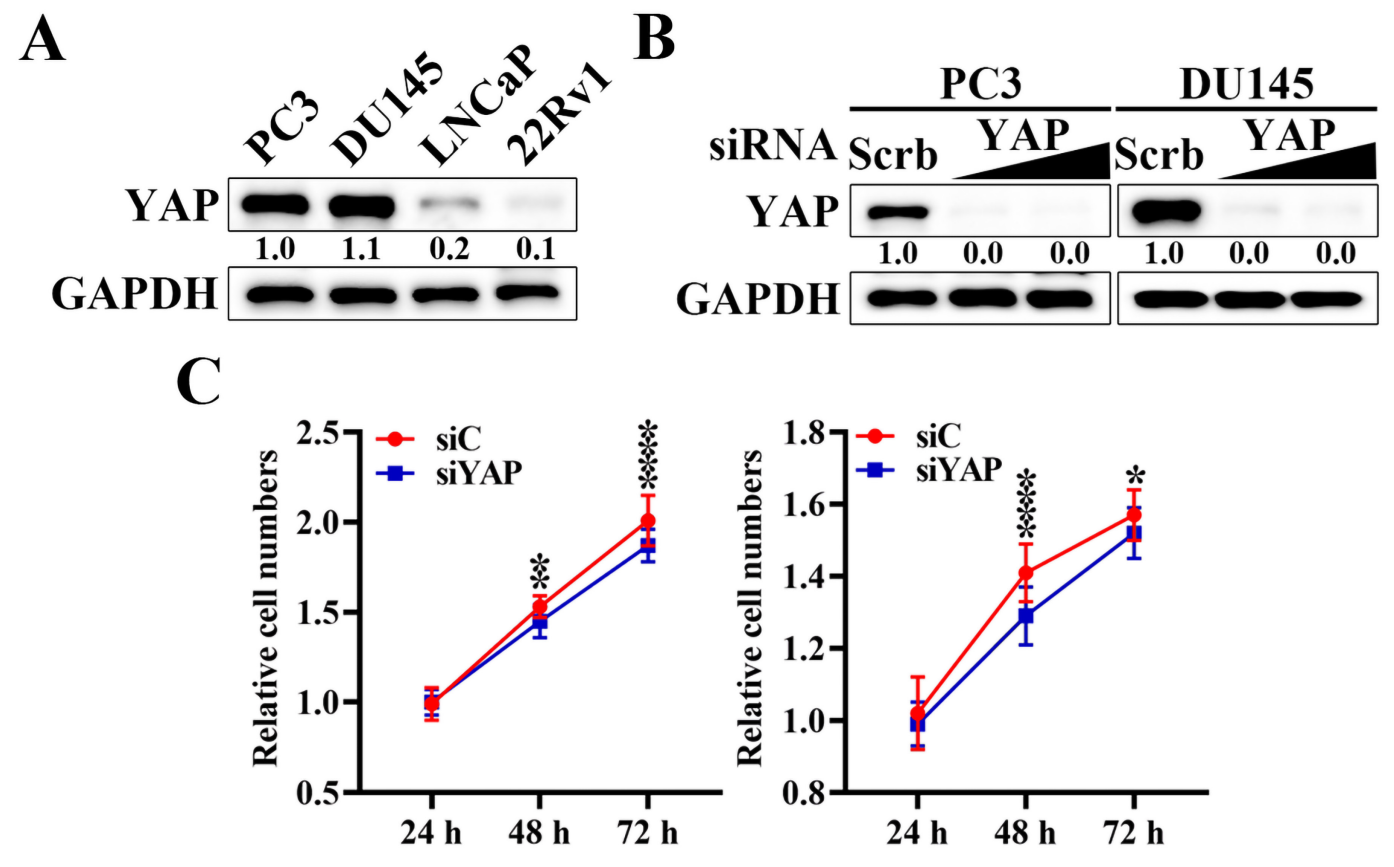
** Inhibition of YAP expression suppresses the proliferation of prostate cancer cells.** (A) Western blot analysis of the differential expression level of YAP protein in human prostate cancer cell lines: PC3, DU145, LNCaP, and 22Rv1 cells. (B) Western blot analysis of the effect of siRNA-mediated knockdown of YAP by siRNA in PC3 and DU145 cells. Cells were transfected with increasing concentrations of YAP-specific siRNA or scrambled (Scrb) control siRNA for 72 hours. Relative expression levels were quantified by densitometric analysis using ImageJ and normalized to GAPDH. (C) Cell proliferation assays were performed in PC3 (left) and DU145 (right) cells after YAP knockdown. Cells were transfected with scrambled siRNA (siScrb) or YAP siRNA (siYAP), and cell numbers were assessed at 24, 48, and 72 hours by MTT assays. Data are presented as mean ± S.D. (n = 3), * *p* < 0.05, ** *p* < 0.01, **** *p* < 0.0001 compared with the corresponding siC (siScrb) group.

To explore the oncogenic potential of YAP in prostate cancer, we first examined the expression of YAP across a panel of human prostate cancer cell lines, including PC3, DU145, LNCaP, and 22Rv1 cells. Western blot analysis revealed that YAP protein levels were markedly higher in PC3 and DU145 cells compared with LNCaP and 22Rv1 cells (Figure 1A). Based on their elevated YAP expression levels and androgen-independent characteristics, PC3 and DU145 cells were selected as representative castration-resistant prostate cancer models for subsequent mechanistic and functional analyses. Transfection with YAP-specific siRNA resulted in a significant reduction in YAP protein expression relative to scrambled siRNA (Figure 1B). Functionally, YAP silencing significantly inhibited cell proliferation in PC3 (Figure 1C left) and DU145 (Figure 1C right) cells, as shown by reduced cell numbers at 48 hours and 72 hours compared with control siRNA groups. Collectively, these findings indicate that YAP is aberrantly expressed in prostate cancer cells and plays a critical role in sustaining the proliferative capacity.

### ATN selectively suppresses the viability of CRPC cells by promoting Hippo pathway activation and YAP degradation

**Figure 2:**
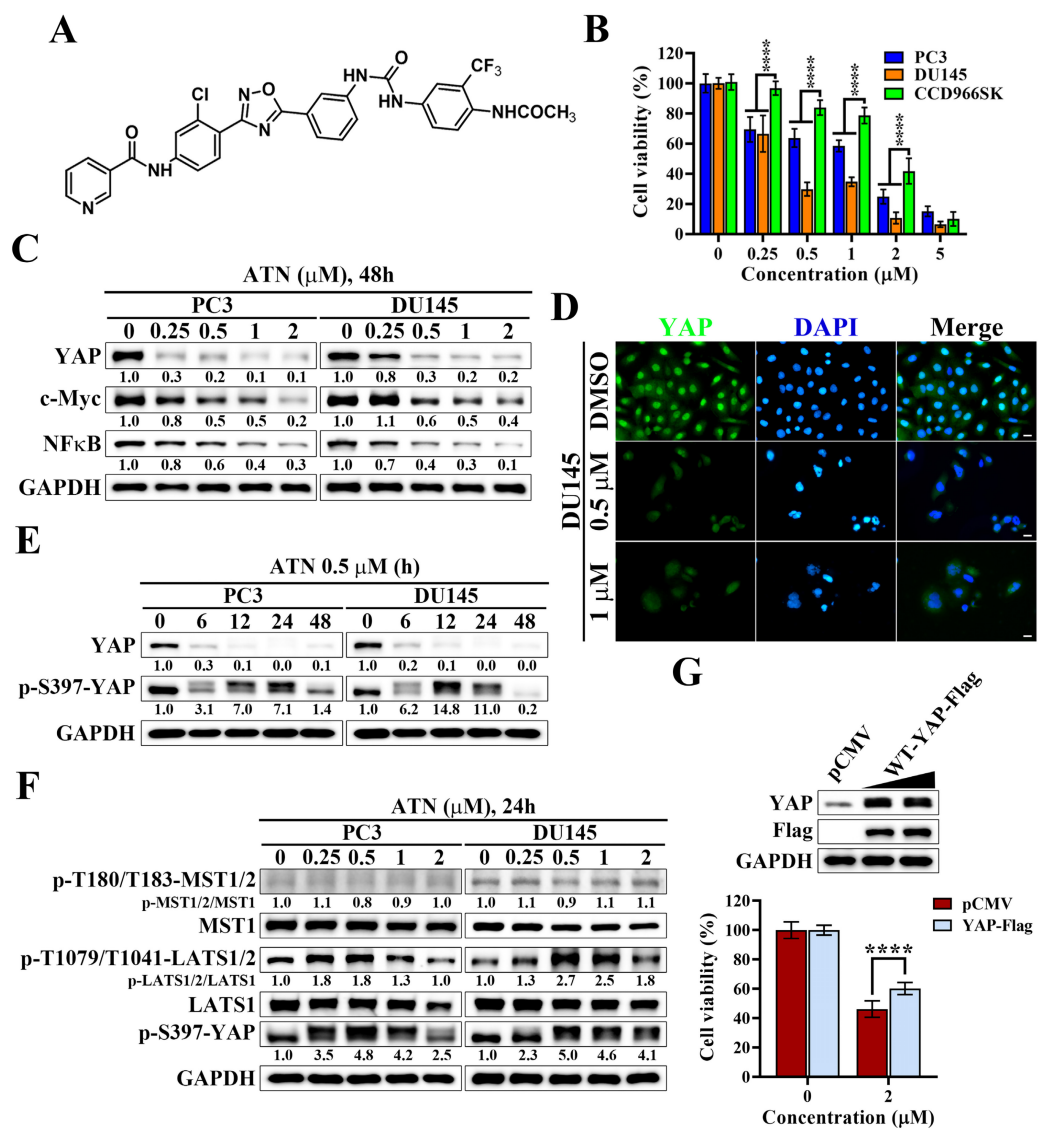
** Suppression of YAP signaling by YAP inhibitor ATN inhibits the cell viability of CRPC cells.** (A) Chemical structure of ATN. (B) Differential suppressive effects of ATN on cell viability in PC3, DU145, and CCD966SK cells. (C) Western blot analysis of the effect of ATN on YAP, c-Myc, and NFκB expression in PC3 and DU145 cells following 48 hours of treatment with ATN at the indicated concentrations. (D) Immunofluorescence staining showing ATN-induced reduction of YAP (green) nuclear signal in DU145 cells after treatment with 0.5 μM or 1 μM ATN for 24 hours. Nuclei were counterstained with DAPI (blue). Scale bars, 10 μm. (E) Western blot analysis of the time-dependent effects of ATN (0.5 μM) on YAP and p-S397-YAP expression in PC3 and DU145 cells. (F) Western blot analysis of the effect of ATN on Hippo pathway kinases MST1/2, LATS1/2, and their phosphorylation, and YAP phosphorylation in PC3 and DU145 cells treated with indicated concentrations of ATN for 24 hours. (G) Overexpression of YAP-Flag rescues ATN-induced cytotoxicity. Western blot analysis confirms ectopic expression of pCMV vector and YAP-Flag in PC3 cells (upper panel), and cell viability was assessed after 72 hours of ATN (2 μM) treatment (lower panel). Data are represented as the means ± S.D. (n = 3), **** *p* < 0.0001.

Since YAP was found to be highly expressed and required for proliferation in prostate cancer cells, we next evaluated whether ATN (Figure 2A), a YAP-targeting compound synthesized in our laboratory, could suppress the growth of CRPC cells. As shown in Figure 2B, ATN treatment significantly reduced cell viability in PC3 and DU145 cells in a concentration-dependent manner, with IC₅₀ values of 1.13 μM and 0.43 μM, respectively. In contrast, human skin fibroblast CCD966SK cells exhibited lower sensitivity to ATN, with an IC₅₀ value of 1.72 μM (Figure 2B). To further evaluate the selectivity of ATN, the selectivity index (SI) was calculated as the ratio of IC₅₀ in CCD966SK cells to that in cancer cells. The SI values were approximately 1.52 for PC3 cells and 4.00 for DU145 cells, indicating preferential cytotoxicity of ATN toward CRPC cells, particularly DU145 cells. To investigate the underlying mechanism, we examined the effect of ATN on YAP signaling. Western blot analysis demonstrated that ATN significantly decreased YAP protein levels in both CRPC cell lines and downregulated its oncogenic downstream effectors, including c-Myc 29, 30 and NFκB 31, 32 (Figure 2C). Consistently, immunofluorescence analysis revealed a pronounced reduction in nuclear YAP fluorescence intensity following ATN treatment in DU145 cells (Figure 2D), indicating that ATN effectively decreases YAP protein abundance in CRPC cells. Time-course experiments further showed that ATN induced YAP phosphorylation at Ser397, a modification known to promote its cytoplasmic retention and proteasomal degradation, resulting in progressive depletion of YAP protein over time (Figure 2E). Consistent with our previous studies 27, ATN enhanced phosphorylation of upstream Hippo pathway kinases, LATS kinases (LATS1/2 at Thr1079/Thr1041), together with increased YAP phosphorylation at Ser397, indicating activation of the Hippo signaling cascade and functional inactivation of YAP in prostate cancer cells (Figure 2F). To determine whether YAP suppression is functionally involved in ATN-induced cytotoxicity, rescue experiments were performed by ectopic expression of YAP. Overexpression of YAP-Flag partially restored cell viability in ATN-treated PC3 cells, recovering approximately 35% of viability relative to the vector control (Figure 2G). These results demonstrate that ATN selectively inhibits CRPC cell viability by activating the Hippo pathway, promoting YAP phosphorylation and degradation, and suppressing YAP-dependent oncogenic signaling.

### ATN inhibits proliferation and induces apoptosis in CRPC cells

**Figure 3:**
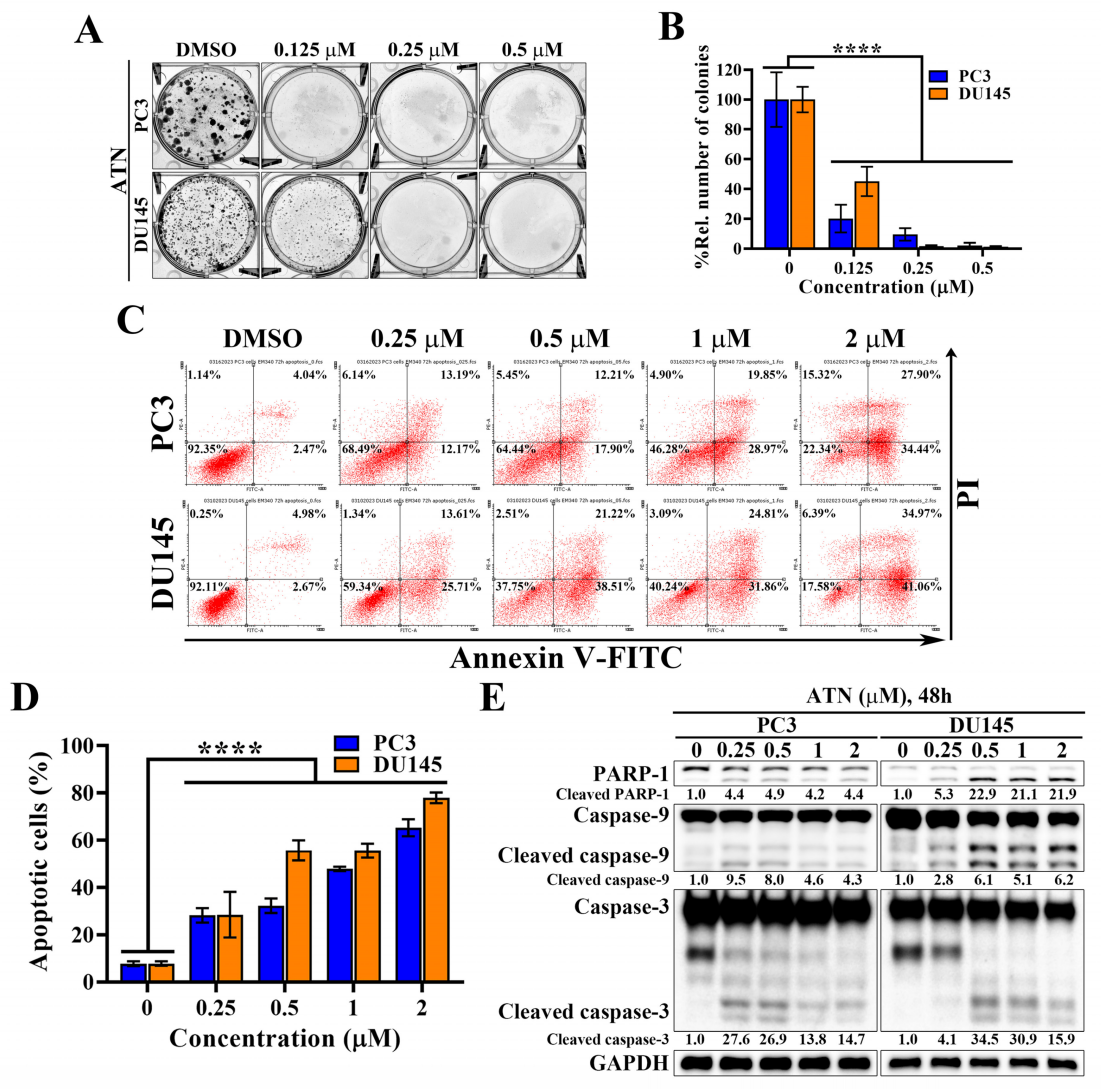
** Anti-proliferative and pro-apoptotic activities of ATN against CRPC cells.** (A) Representative images showing the effects of ATN on colony formation in PC3 and DU145 cells. (B) Quantitative results of colony formation expressed as the percentage relative to the DMSO-treated control. Data are represented as the means ± S.D. (n = 3), **** *p* < 0.0001. (C) Representative flow cytometry plots showing the effects of ATN on apoptosis in PC3 and DU145 cells. Annexin V-FITC/propidium iodide double staining of cells after treatment with ATN at the indicated concentrations for 72 hours. The percentages shown in each quadrant indicate the proportion of cells within that population. The apoptotic cells were defined as the sum of Annexin V-FITC positive/PI negative and Annexin V-FITC positive/PI positive populations. (D) Quantitative analysis of apoptotic cell populations in PC3 and DU145 cells, expressed as a percentage relative to the DMSO-treated control. Data are represented as the means ± S.D. (n = 3), **** *p* < 0.0001. (E) Western blot analysis of the effect of ATN on apoptosis-related proteins, including PARP-1 cleavage and activation of caspase 3 and caspase 9, in PC3 and DU145 cells.

Since ATN markedly suppressed CRPC cell viability through Hippo pathway activation and YAP degradation, we next evaluated its effects on proliferation in CRPC cells. Colony formation assays revealed that ATN significantly reduced clonogenic survival of PC3 and DU145 cells in a concentration-dependent manner, with near-complete inhibition observed at 0.25 and 0.5 μM (Figure 3A and 3B). To further explore whether the reduction in cell viability was attributable to apoptosis, Annexin V-FITC/propidium iodide (PI) double staining was conducted and then analyzed by flow cytometry. Flow cytometry analysis demonstrated a concentration-dependent increase in apoptotic cell populations in both PC3 and DU145 cells following ATN treatment (Figure 3C). Quantification confirmed that apoptosis rates rose progressively, reaching approximately 50% at 1 μM in both DU145 and PC3 cells (Figure 3D). Consistent with these findings, ATN treatment triggered activation of the apoptotic pathway, as evidenced by PARP-1 cleavage and activation of caspase-9 and caspase-3 (Figure 3E). Together, these results reveal that ATN-mediated growth inhibition is associated with reduced proliferation and induction of apoptotic cell death in CRPC cells.

### ATN suppresses cancer stem-like properties in CRPC cells

**Figure 4:**
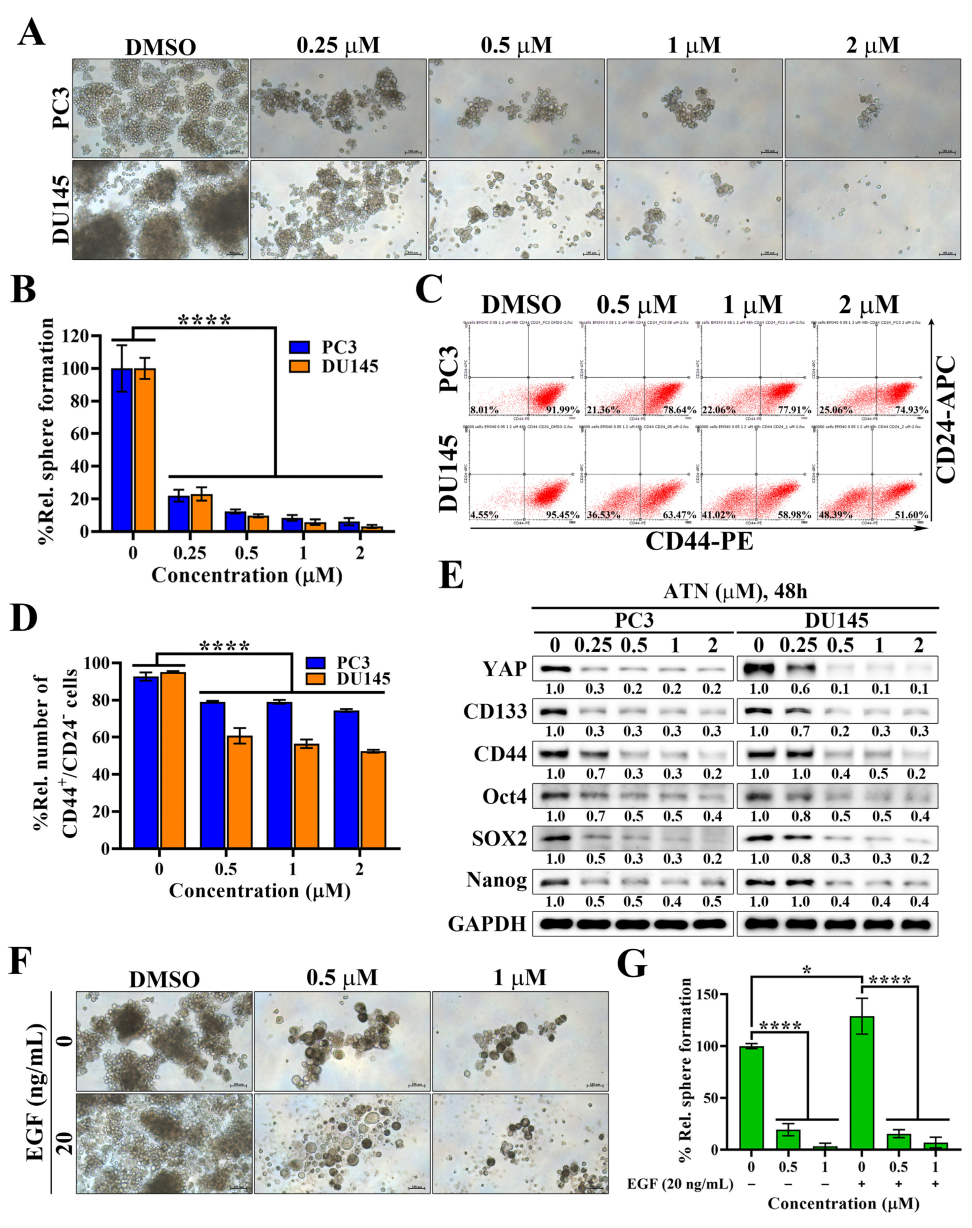
** Evidence that ATN inhibits prostate cancer stem cells.** (A) Representative images showing the effect of ATN on tumorsphere formation in PC3 and DU145 cells (100× magnification). Scale bars, 100 μm. (B) Quantitative results of the effect of ATN on tumorsphere formation expressed as the percentage relative to the DMSO-treated control. Data are represented as the means ± S.D. (n = 4), **** *p* < 0.0001. (C) Representative results showing the effects of ATN on CD44⁺/CD24⁻ prostate CSCs populations assessed by flow cytometry in PC3 and DU145 cells following 48 hours of treatment with ATN. The percentages shown in quadrants indicate the proportion of cells within that population. (D) Quantitative results of the effect of ATN on CD44⁺/CD24⁻ prostate CSCs populations expressed as the percentage relative to the DMSO-treated control. Data are represented as the means ± S.D. (n = 3), **** *p* < 0.0001. (E) Western blot analyses of the effect of ATN on the expression of cancer stem cell-related proteins, including CD133, CD44, Oct4, SOX2, and Nanog, in PC3 and DU145 cells following 48 hours of treatment with ATN at the indicated concentrations. (F) Representative images showing the suppressive effect of ATN on EGF-induced tumorsphere formation in PC3 cells (100× magnification). Scale bars, 100 μm. (G) Quantitative results of the effect of ATN on the tumorsphere formation shown in (F) expressed as the percentage relative to the DMSO-treated control. Data are represented as the means ± S.D. (n = 4), * *p* < 0.05, **** *p* < 0.0001.

Cancer stem cells (CSCs) play a pivotal role in therapeutic resistance, disease recurrence, and metastatic progression of CRPC 33, 34. To investigate whether ATN could target this malignant subpopulation, we first performed tumorsphere formation assays (Figure 4A). ATN markedly suppressed the tumorsphere formation capacity of PC3 and DU145 cells in a concentration-dependent manner, with near-complete inhibition at the concentration of 1 μM (Figure 4A and 4B). Flow cytometric analysis further revealed that ATN significantly reduced the proportion of CD44⁺/CD24⁻ subpopulations, a hallmark of prostate CSCs (Figure 4C and 4D). Previous studies have demonstrated that EGFR-mediated signaling drives the propagation of prostate CSCs, while SOX2 serves as a key regulator of EGFR-dependent self-renewal 22, 35. Western blotting analysis demonstrated that ATN significantly downregulated cancer stemness-associated proteins, including CD133, CD44, Oct4, SOX2, and Nanog, together with reduced YAP expression (Figure 4E). To further examine the effect of ATN on EGFR signaling-mediated activation of cancer stemness in prostate cancer cells, we assessed tumorsphere formation in the presence of EGF stimulation. As expected, EGF promoted the growth of prostate CSCs in control cells; however, ATN treatment strongly suppressed EGF-induced tumorsphere formation (Figure 4F and 4G). Collectively, these findings demonstrate that ATN impairs CSC-associated phenotypes in CRPC, highlighting its therapeutic potential to overcome cancer stemness-driven progression in advanced prostate cancer.

### ATN inhibits migration and EMT-associated protein expression in CRPC cells

**Figure 5:**
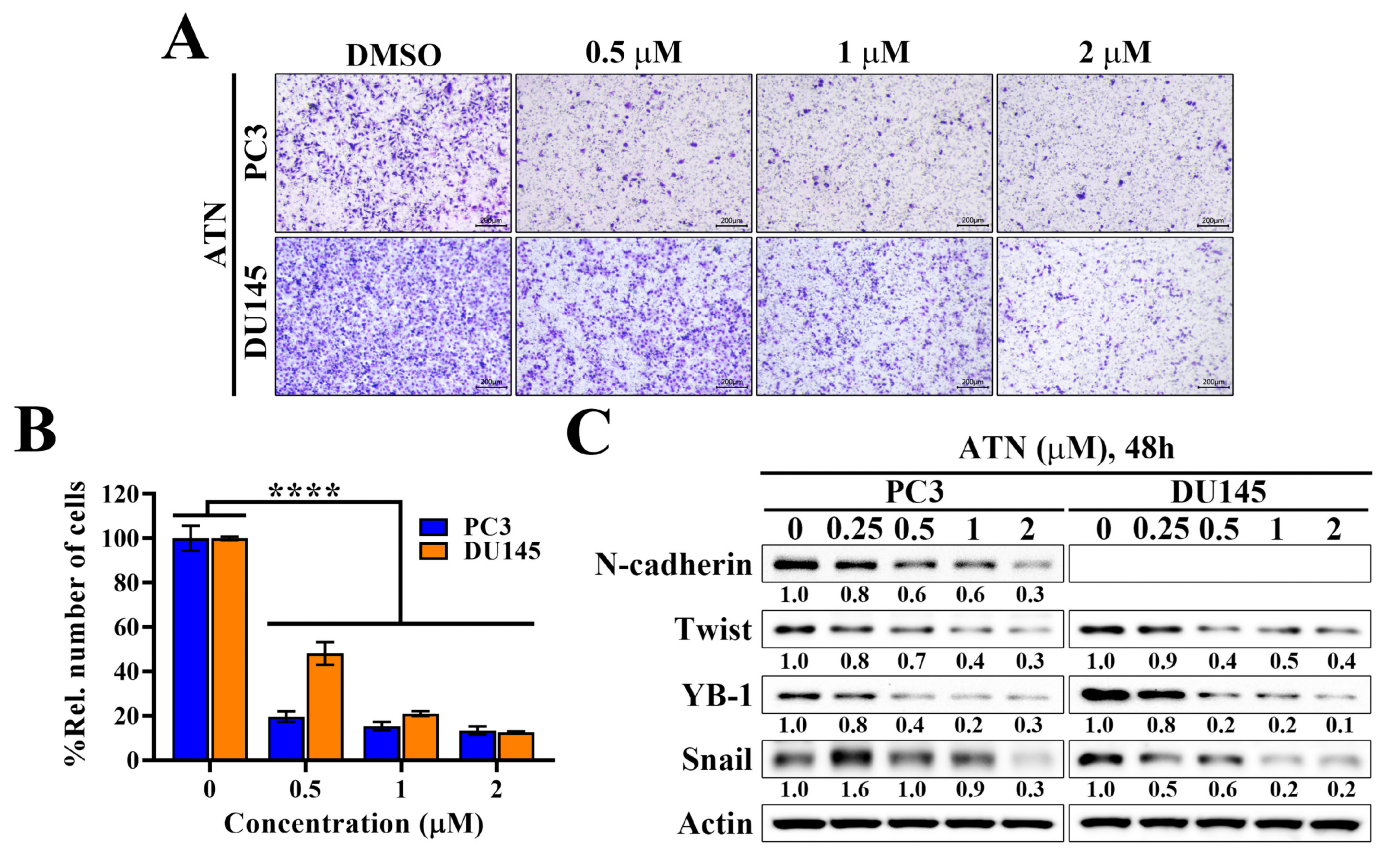
** Evidence that suppression of YAP expression by ATN inhibits the migration of CRPC cells.** (A) Representative images showing the concentration-dependent effects of ATN on migration by transwell migration assays in PC3 and DU145 cells. (B) Quantitative results of the effect of ATN on migration. Relative number of migrated cells were expressed as the percentage compared with the DMSO control. Data are represented as the means ± S.D. (n = 3), **** *p* < 0.0001. (C) Western blot analyses of the effect of ATN on the expressions of migration-related proteins, including N-cadherin, Twist, YB-1, and Snail, in PC3 and DU145 cells.

CSCs not only maintain tumor-initiating capacity but also enhance EMT, a process tightly linked to metastatic dissemination in CRPC. Notably, EGFR signaling has been reported to be a major inducer of EMT, acting through downstream pathways such as ERK and Akt to upregulate mesenchymal markers and transcriptional regulators 36, 37. To investigate whether ATN affects this malignant phenotype, we performed transwell migration assays and the results showed that ATN markedly suppressed the migratory capacity of PC3 and DU145 cells in a concentration-dependent manner, with significant inhibition at 0.5 μM and almost complete blockade at 2 μM (Figure 5A and 5B). Moreover, Western blot analysis showed that ATN downregulated the mesenchymal marker N-cadherin and reduced the expressions of key EMT-associated transcriptional factors Twist, YB-1, and Snail in both CRPC cell lines (Figure 5C). These findings indicated that, in addition to impairing CSC-driven self-renewal, ATN might also abrogate EGFR-induced EMT programs, thereby inhibiting CRPC cell migration. Collectively, these data demonstrate that ATN targets multiple malignant features of CRPC, including proliferation, cancer stemness, and EMT-driven metastatic potential.

### ATN disrupts the YAP-associated EGFR signaling and suppresses downstream MEK/ERK and Akt/mTOR signaling in CRPC cells

**Figure 6:**
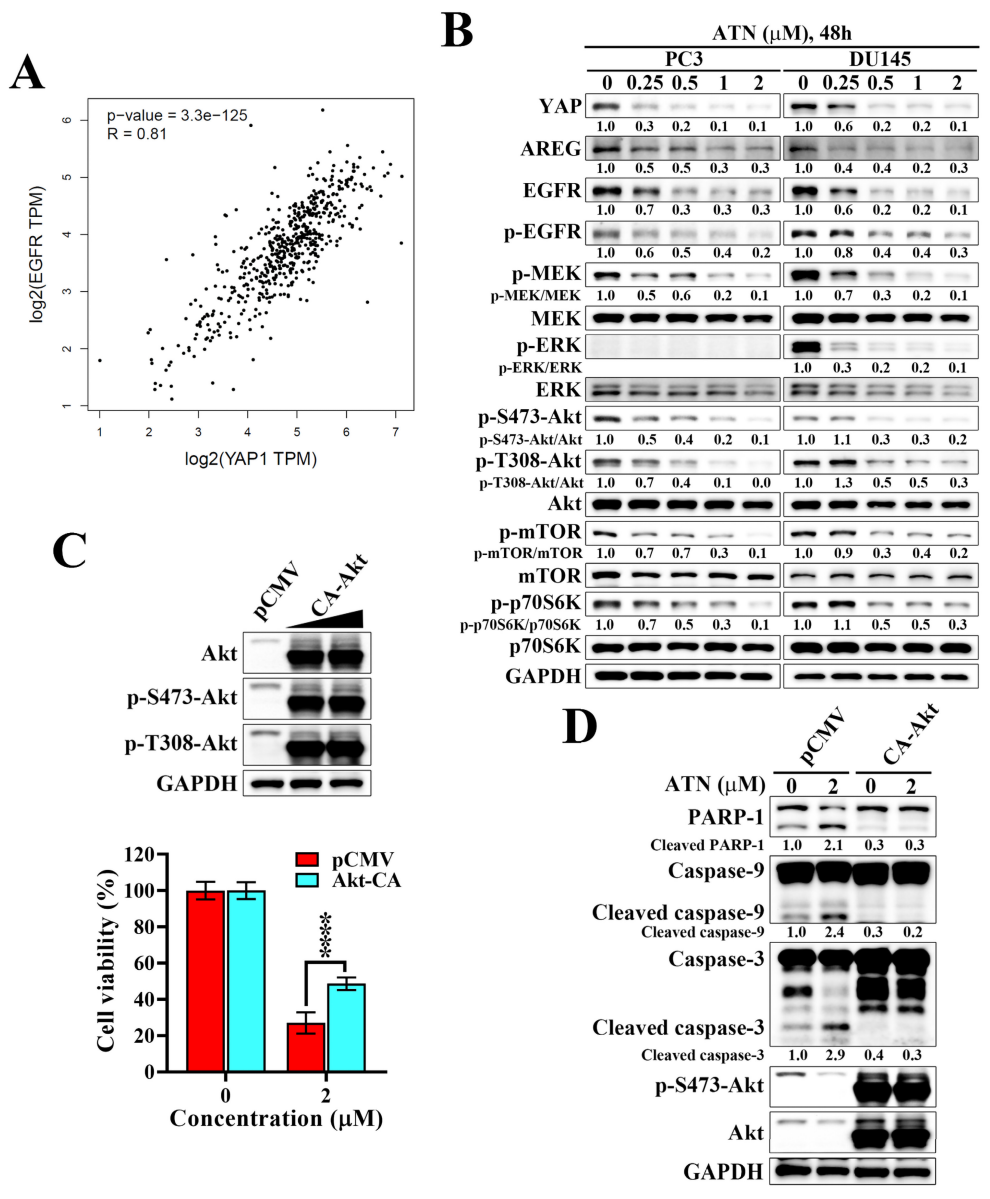
** ATN suppresses the YAP-associated EGFR/Akt signaling in CRPC cells.** (A) Correlation analysis of YAP and EGFR mRNA expression based on TCGA prostate cancer dataset, showing a significant positive correlation (Spearman's rank correlation coefficient r = 0.81, *p* = 3.3×10^-125^). (B) Western blot analyses of the effect of ATN on the expressions of AREG, EGFR, and downstream signaling proteins, including phosphorylated and total levels of MEK, ERK, Akt, mTOR, and p70S6K, in PC3 and DU145 cells treated with the indicated concentrations of ATN for 48 hours. (C) Upper panel, Western blot validation of Akt and p-Akt expression levels in PC3 cells ectopically expressed the pCMV vector or constitutively active Akt (CA-Akt). Lower panel, cell viability of PC3 cells transfected with pCMV or CA-Akt and treated with ATN was determined by MTT assay. Data are represented as the means ± S.D. (n = 4), **** *p* < 0.0001. (D) Western blot analysis of apoptotic markers, including PARP-1, caspase-9, cleaved caspase-9, caspase-3, and cleaved caspase-3, in PC3 cells transfected with pCMV or CA-Akt and treated with ATN for 48 hours.

Analysis of the Cancer Genome Atlas prostate adenocarcinoma (TCGA-PRAD) dataset revealed a strong positive correlation between YAP and EGFR expression (Spearman's rank test r = 0.81, *p* = 3.3×10^-125^), supporting the existence of a functional association between YAP and EGFR in prostate cancer (Figure 6A). Amphiregulin (AREG), a well-established transcriptional target of YAP and ligand of EGFR, is frequently overexpressed in aggressive tumors and functions as an autocrine stimulator of EGFR signaling 38, 39. We found that ATN treatment in PC3 and DU145 cells led to a marked reduction in YAP, EGFR, and AREG protein levels, accompanied by decreased phosphorylation of EGFR and suppression of downstream MEK/ERK and Akt/mTOR/p70S6K signaling cascades (Figure 6B). To establish the functional importance of AREG/EGFR inhibition, we performed rescue experiments using constitutively active Akt (CA-Akt). Forced Akt activation restored phosphorylation of Akt at S473 and T308, and significantly counteracted ATN-induced cytotoxicity, resulting in approximately 78% recovery of cell viability compared with the vector control (Figure 6C). Moreover, CA-Akt expression diminished ATN-induced apoptosis, as shown by reduced cleavage of PARP-1, caspase-9, and caspase-3 (Figure 6D). Together, these findings demonstrate that ATN suppresses CRPC cell survival by disrupting the YAP-associated EGFR signaling and its downstream Akt/mTOR pathway, providing mechanistic insight into how ATN attenuates cell viability of CRPC.

## Discussion

Prostate cancer remains one of the most prevalent malignancies in men, and progression to CRPC represents a major therapeutic challenge. In the present study, we identified a novel oxadiazole-based compound, ATN, that exhibits potent anti-cancer activity in CRPC cellular models through activation of the Hippo pathway and destabilization of YAP. Our findings collectively suggest that ATN exerts a coordinated suppression of multiple oncogenic processes, including proliferation, survival, cancer stem-like properties, and migration, which are hallmarks of CRPC progression.

The Hippo-YAP pathway has emerged as a critical regulator of tumorigenesis, cancer stemness, and therapeutic resistance across multiple malignancies 40. Pharmacological inhibition of YAP has therefore been widely pursued; however, most currently available YAP-targeting strategies face significant limitations. Verteporfin, the most extensively studied YAP inhibitor, disrupts YAP-TEAD transcriptional activity and reduces tumor growth in various models 41. However, verteporfin suffers from important limitations, including phototoxicity, poor bioavailability, and off-target effects that limit its clinical applicability. Other experimental YAP inhibitors have targeted upstream Hippo kinases, disrupted YAP-TEAD binding, or modulated YAP nuclear localization, but most lack selectivity or sufficient potency for clinical translation. In contrast, ATN operates through a distinct mechanism by reactivating upstream Hippo kinases (LATS1/2), leading to YAP phosphorylation at Ser397 and subsequent proteasomal degradation. This mode of action represents an upstream regulatory strategy that may provide more sustained suppression of YAP signaling compared with agents that solely interfere with transcriptional activity. Another key difference between ATN and existing YAP inhibitors lies in its integrated regulation of oncogenic signaling beyond YAP. Our data showed that ATN simultaneously downregulated the expression of c-Myc and NFκB, which are well-established downstream targets of YAP and critical regulators of proliferation, apoptosis resistance, and inflammation in prostate cancer. Furthermore, ATN markedly suppressed cancer stem-like properties and EMT, both of which are closely linked to therapeutic resistance and metastatic progression in CRPC. These findings suggest that targeting YAP through Hippo pathway activation may exert broader anti-tumor effects than previously appreciated.

A particularly novel and significant aspect of this study is the demonstration that ATN disrupts YAP-associated EGFR signaling. Accumulating evidence indicates that YAP transcriptionally induces AREG, which functions as an autocrine ligand to activate EGFR signaling and amplify downstream oncogenic cascades 39, 42. This coordinated signaling interaction provides a mechanism by which YAP sustains EGFR signaling, promoting uncontrolled growth, cancer stemness, and therapy resistance. Our findings extend this mechanistic framework to CRPC, demonstrating that ATN reduces YAP stability, suppresses AREG expression, and attenuates EGFR activation, thereby disrupting this oncogenic signaling axis. Importantly, beyond its effects on ligand production, ATN also significantly reduces total EGFR protein levels, indicating that it targets both upstream transcriptional regulation and receptor availability. This dual effect has important mechanistic implications, as downregulation of EGFR constrains ligand-dependent pathway reactivation and reinforces suppression of EGFR signaling. Collectively, this supports a model in which ATN exerts a coordinated blockade of the YAP-AREG-EGFR axis, rather than acting solely through modulation of ligand availability. Notably, the interplay between YAP and RTK signaling appears to be more complex than a unidirectional pathway. Emerging evidence suggests that RTK signaling can also reinforce YAP activity, forming a reciprocal regulatory network that contributes to therapy resistance. For example, TNIK has been shown to phosphorylate EGFR and promote CRPC progression under androgen-deprived conditions 21, supporting the concept that persistent RTK signaling is a driver of therapy resistance in advanced prostate cancer. Furthermore, AXL has been reported to activate YAP through the EGFR-LATS1/2 axis and to confer resistance to EGFR-targeted therapies, providing a broader mechanistic framework for RTK-mediated YAP reactivation 43. Thus, our findings in CRPC are consistent with an emerging model in which YAP not only regulates EGFR signaling output but also participates in reciprocal RTK-driven adaptive resistance networks. In this context, ATN-mediated degradation of YAP may disrupt both ligand-dependent EGFR activation and broader RTK-driven resistance mechanisms. The limited clinical success of EGFR-targeted therapies in prostate cancer further underscores the importance of this upstream regulatory strategy. Although EGFR signaling is frequently upregulated in CRPC, inhibitors targeting EGFR have shown modest efficacy in clinical settings. This failure has been attributed to multiple mechanisms, including pathway redundancy, compensatory activation of PI3K/Akt and MAPK signaling, and persistent ligand-driven autocrine activation. Our data suggest that targeting YAP may circumvent these limitations by reducing AREG production and suppressing EGFR signaling at its source. By simultaneously suppressing YAP and EGFR-driven signaling, ATN may provide a dual blockade that is less susceptible to compensatory feedback activation. This concept is consistent with recent studies in other RTK-driven cancers showing that YAP reactivation can function as a bypass mechanism during EGFR-targeted therapy 43.

From a translational perspective, these findings position ATN as a promising candidate for combination therapy in CRPC. Given the complexity and redundancy of oncogenic signaling networks in advanced prostate cancer, targeting a single pathway is often insufficient to achieve durable responses. The ability of ATN to simultaneously inhibit YAP-driven transcriptional programs, EGFR signaling, cancer stem-like properties, and EMT suggests that it may enhance the efficacy of existing therapies, including androgen receptor inhibitors, PI3K/Akt pathway inhibitors, and immunotherapeutic approaches.

Despite these promising findings, several limitations of the present study should be acknowledged. First, the current work is based exclusively on *in vitro* cellular models and does not address the *in vivo* anti-tumor efficacy, pharmacokinetics, or safety profile of ATN. Future studies employing appropriate *in vivo* models, such as CRPC xenografts or patient-derived tumor models, will be essential to validate the therapeutic potential of ATN and to assess its bioavailability, toxicity, and translational feasibility. Second, although ATN demonstrated preferential cytotoxicity toward CRPC cells compared with skin fibroblasts, the evaluation of compound selectivity remains limited by the use of a non-prostate-derived normal cell line. The inclusion of normal prostate epithelial cells, such as RWPE-1, would provide a more physiologically relevant assessment of tissue-specific toxicity and therapeutic window. Future investigations incorporating normal prostate epithelial models will be essential to further validate the selectivity and safety profile of ATN. Third, although our data support disruption of the YAP-AREG-EGFR axis, further studies are required to determine whether AREG suppression is directly mediated by YAP at the transcriptional level. Fourth, the potential effects of ATN on TAZ or other parallel signaling pathways warrant further investigation. Finally, structural optimization of ATN may improve its potency and pharmacokinetic properties, thereby facilitating its progression toward preclinical development.

In conclusion, this study identifies ATN as a novel Hippo pathway activator that exerts multi-faceted anti-cancer effects in CRPC. By promoting YAP degradation and disrupting the YAP-associated EGFR signaling network, ATN suppresses key oncogenic processes, including proliferation, cancer stem-like properties, EMT, and survival signaling. These findings not only advance our understanding of Hippo-YAP biology in prostate cancer but also provide a strong rationale for targeting the YAP-EGFR axis as a therapeutic strategy in CRPC.

## Data Availability

All the data of this research are available upon reasonable request.

## Abbreviations

CRPC: castration-resistant prostate cancer
YAP: Yes-associated protein
ADT: androgen deprivation therapy
EGFR: epidermal growth factor receptor
EMT: epithelial-mesenchymal transition
AREG: amphiregulin
FBS: fetal bovine serum
MST1/2: mammalian Ste20-like kinases 1/2
MOB1A/B: MOB kinase activator 1A/B
LATS1/2: large tumor suppressor kinase 1/2
TAZ: transcriptional coactivator with a PDZ-binding motif
TEAD: transcriptional enhanced associate domain
